# Supplementary material for: Single Cell Transcriptomic and Chromatin Profiles Suggest Layer Vb Is the Only Layer With Shared Excitatory Cell Types in the Medial and Lateral Entorhinal Cortex
Source: Front Neural Circuits. 2022 Jan 26;15:806154. doi: 10.3389/fncir.2021.806154 (PMC8826650; doi:10.3389/fncir.2021.806154)
Supplement: Supplementary file 14 [file Data_Sheet_1.ZIP › Sup_vs_Deep_Motifs/homerResults/motif17.similar.html]

motif17

## Information for motif17

A
T
G
C
A
C
T
G
A
C
T
G
G
A
C
T
A
T
G
C
T
C
G
A
G
A
C
T
C
A
G
T
  
Reverse Opposite:  

G
T
C
A
C
T
G
A
A
G
C
T
A
T
C
G
C
T
G
A
A
G
T
C
G
T
A
C
T
A
C
G
  

|  |  |
| --- | --- |
| p-value: | 1e-42 |
| log p-value: | -9.782e+01 |
| Information Content per bp: | 1.797 |
| Number of Target Sequences with motif | 1377.0 |
| Percentage of Target Sequences with motif | 4.11% |
| Number of Background Sequences with motif | 238.4 |
| Percentage of Background Sequences with motif | 2.79% |
| Average Position of motif in Targets | 382.4 +/- 264.5bp |
| Average Position of motif in Background | 368.6 +/- 319.8bp |
| Strand Bias (log2 ratio + to - strand density) | 0.1 |
| Multiplicity (# of sites on avg that occur together) | 1.02 |
| Motif File: | file (matrix) reverse opposite |

### Similar de novo motifs found

|  |  |  |  |  |  |  |  |
| --- | --- | --- | --- | --- | --- | --- | --- |
| Rank | Match Score | Redundant Motif | P-value | log P-value | % of Targets | % of Background | Motif file |
| 1 | 0.769 | A C G T A C T G A G C T A T C G A C T G A G C T A G T C C G T A A C G T A C G T | 1e-38 | -87.733900 | 0.50% | 0.16% | motif file (matrix) |
| 2 | 0.645 | A C T G A G T C A C G T A C T G C G T A A G T C A G T C A C T G | 1e-14 | -33.771451 | 0.25% | 0.09% | motif file (matrix) |
